# Use of antenatal corticosteroids at health facilities and communities in low-and-middle income countries

**DOI:** 10.1186/s12978-016-0176-2

**Published:** 2016-05-27

**Authors:** Mabel Berrueta, Jennifer Hemingway-Foday, Vanessa R. Thorsten, Robert L. Goldenberg, Waldemar A. Carlo, Ana Garces, Archana Patel, Sarah Saleem, Omrana Pasha, Elwyn Chomba, Patricia L. Hibberd, Nancy F. Krebs, Shivaprasad Goudar, Richard J. Derman, Fabian Esamai, Edward A Liechty, Janet L. Moore, Elizabeth M. McClure, Marion Koso-Thomas, Pierre M. Buekens, José M. Belizán, Fernando Althabe

**Affiliations:** Institute for Clinical Effectiveness and Health Policy (IECS), Buenos Aires, Argentina; RTI International, Durham, NC USA; Department of Obstetrics and Gynecology, Columbia University, New York, NY USA; University of Alabama at Birmingham, Birmingham, AL USA; Fundación para la Alimentación y Nutrición de Centro América y Panamá, Guatemala City, Guatemala; Lata Medical Research Foundation, Indira Gandhi Government Medical College, Nagpur, India; Department of Community Health Sciences, Aga Khan University, Karachi, Pakistan; University Teaching Hospital, Lusaka, Zambia; Massachusetts General Hospital, Boston, MA USA; University of Colorado School of Medicine, Denver, CO USA; KLE University’s Jawaharlal Nehru Medical College, Belgaum, Karnataka India; Christiana Health Care, Newark, DE USA; Moi University School of Medicine, Eldoret, Kenya; School of Medicine, Indiana University, Indianapolis, IN USA; Eunice Kennedy Shriver National Institute of Child Health and Human Development, Bethesda, MD USA; Tulane School of Public Health & Tropical Medicine, New Orleans, LA USA

## Abstract

**Background:**

Antenatal corticosteroids (ACS) for women at high risk of preterm birth is an effective intervention to reduce neonatal mortality among preterm babies delivered in hospital settings, but has not been widely used in low-middle resource settings. We sought to assess the rates of ACS use at all levels of health care in low and middle income countries (LMIC).

**Methods:**

We assessed rates of ACS in 7 sites in 6 LMIC participating in the Eunice Kennedy Shriver National Institute of Child Health and Human Development’s Global Network for Women and Children’s Health Research Antenatal Corticosteroids Trial (ACT), a cluster-randomized trial to assess the feasibility, effectiveness, and safety of a multifaceted intervention designed to increase the use of ACS. We conducted this analysis using data from the control clusters, which did not receive any components of the intervention and intended to follow usual care. We included women who delivered an infant with a birth weight <5th percentile, a proxy for preterm birth, and were enrolled in the Maternal Newborn Health (MNH) Registry between October 2011 and March 2014 in all clusters. A survey of the site investigators regarding existing policies on ACS in health facilities and for health workers in the community was part of pre-trial activities.

**Results:**

Overall, of 51,523 women delivered in control clusters across all sites, the percentage of <5th percentile babies ranged from 3.5 % in Kenya to 10.7 % in Pakistan. There was variation among the sites in the use of ACS at all hospitals and among those hospitals having cesarean section and neonatal care capabilities (bag and mask and oxygen or mechanical ventilation). Rates of ACS use for <5th percentile babies in all hospitals ranged from 3.8 % in the Kenya sites to 44.5 % in the Argentina site, and in hospitals with cesarean section and neonatal care capabilities from 0 % in Zambia to 43.5 % in Argentina. ACS were rarely used in clinic or home deliveries at any site. Guidelines for ACS use at all levels of the health system were available for most of the sites.

**Conclusion:**

Our study reports an overall low utilization of ACS among mothers of <5th percentile infants in hospital and clinic deliveries in LMIC.

**Trial Registration:**

clinicaltrials.gov (NCT01084096)

## Background

Preterm birth is a leading cause of neonatal mortality and morbidity [1]. Antenatal corticosteroids (ACS) for pregnant women at high risk of preterm delivery are among the most effective hospital-based interventions in high resource settings to reduce neonatal mortality among preterm newborns [1–7] Despite the burden of preterm-related morbidity and mortality, as well as the effectiveness of ACS, global uptake of this intervention has been relatively low [8–12]. To that purpose, the Eunice Kennedy Shriver National Institute of Child Health and Human Development (NICHD)’s Global Network for Women and Children’s Health Research Antenatal Corticosteroids Trial (ACT) [13–15] assessed the feasibility, effectiveness, and safety of a complex intervention to increase the use of ACS at all levels of care at seven study sites in low and middle-income countries (LMIC) (Argentina, Guatemala, Kenya, Zambia, Pakistan and India [2 sites]). The results showed that the intervention did not significantly reduce neonatal mortality for <5th percentile infants, a proxy for preterm birth, and was associated with an overall 12 % increase in neonatal deaths compared to the control group [16].

The unanticipated ACT results had important implications for policy, practice, and research. The recently released World Health Organization (WHO) guidelines on interventions to improve preterm birth outcomes [17] recommend the use of ACS when several conditions are met: gestational age assessment can be accurately undertaken; preterm birth is considered imminent; there is no clinical evidence of maternal infection; adequate childbirth care is available (including the capacity to recognize and safely manage preterm labor and birth); and the preterm newborn can receive adequate care if needed (including resuscitation, thermal care, feeding support, infection treatment and safe oxygen use). In LMIC’s these preconditions may be met in well-equipped referral hospitals, but are less likely to be met in lower- level facilities, such as second level hospitals and primary health centers, where most of the facility-based deliveries occur in these countries [18]. One of the most important implications for research derived from the ACT results was the need to assess the effectiveness of ACS in lower-level facilities [19, 20].

To define the current patterns of use of ACS for preterm newborns in LMICs is thus important for both practice and research. For large hospitals having cesarean sections capability across 29 LMICs, Vogel et al. [21] reported rates of ACS use ranging from 19 to 91 % among women having preterm births between 26 and 34 weeks gestation. However, the use in lower-level health facilities is still not well known. The ACT trial provides a good source to estimate current use at those settings. The clusters assigned to the control group of the ACT trial received no active intervention, and the data collection procedures depended on an independent health registry that was ongoing since well before the trial started (16). Therefore the use of ACS in the health facilities of these clusters may be a close estimation of the routine use of ACS in these settings.

We sought to determine the rates of ACS use both for health facility and community settings in the clusters assigned to the control group during the ACT trial in six LMICs, aiming to estimate the current use of ACS in preterm births in LMICs.

## Methods

ACT was an 18-month, two-arm, cluster-randomized trial to assess the feasibility, effectiveness, and safety of a multifaceted intervention designed to increase the use of ACS at all levels of health care in LMICs. The trial methods and results are described in detail elsewhere [14, 16]. Briefly, we randomly assigned rural and semi-urban clusters (geographic areas with about 500 births per year) within six countries (Argentina, Guatemala, India [2 sites], Pakistan, Kenya, and Zambia) to standard care or a multifaceted intervention including components to improve identification of women at risk of preterm birth, referral for care, and to facilitate appropriate use of ACS. The primary outcome was 28-day neonatal mortality among infants less than the 5th percentile for birth weight (defined by site-specific data as a proxy for preterm birth). Secondary outcomes included use of ACS, neonatal and perinatal mortality, and suspected maternal infection for all births, irrespective of birth weight.

The outcome data were collected independently by trained registry administrators in a prospective maternal and newborn health (MNH) registry [13], which enrolled and collected outcomes for all pregnant women residing within the study clusters, defined geographic areas which included health facilities. The trial period included births between October 2011 and March 2014, depending on each site’s 18-month enrollment period, with most births occurring in 2012 and 2013.

The clusters assigned to the control group did not receive any training or ACS supplies that were components of the intervention and were intended to follow standard care. Thus the use of ACS in these settings was likely to be similar to routine practice, or perhaps somewhat higher, given that the control clusters were part of the overall ACT project and contamination may have influenced ACS use. In preparation for the trial to help define the standard care for ACS within the health systems, prior to the intervention training activities, each of the study sites conducted a survey of the Ministry of Health and the health facilities serving the study catchment area regarding the existing policies and practice for providing ACS to pregnant women at risk of preterm birth. The survey addressed the use of ACS at hospitals, health clinics as well as use by community health workers outside of formal health facilities.

### Statistical analysis

The aim of this analysis is purely descriptive. We assessed the use of ACS within the control clusters by study site, among the live births with birth weight <5th percentile [16]. Unadjusted frequencies and percentages of characteristics of the control clusters, signs of preterm birth and use of ACS at the individual level are provided. No measures of inference have been calculated, and as such, measures are not adjusted for cluster. We used birth weight rather than gestational age for the main analysis because many women in the registry had missing or uncertain gestational age, ultrasound was often unavailable, and the intervention was designed to improve estimation of gestational age, which could potentially bias gestational age-based analyses. The <5th percentile birth weight group (referred to as <5th percentile babies) was established separately for each site with birth weight data for the pretrial year, in view of the differences in birth weight distributions across the sites. Site-specific cutoffs based on measured weights of live births were 2450 g for Argentina, 2267 g for Guatemala, 2000 g for Belgaum, India, 2000 g for Nagpur, India, 2150 g for Pakistan, 2400 g for Zambia, and 2500 g for Kenya. Babies were classified as <5th percentile on the basis of measured birth weights when available, otherwise, by estimated weights by clinical assessment. Those missing both measured and estimated weights were classified as <5th percentile, since based on historical data from the registry, most of the missing data were for preterm infants. Using this classification, we estimated that 60 % of less-than-5th-percentile births were born at a gestational age younger than 37 weeks in the main study [16].

Just for descriptive purposes, we also estimated the baby’s preterm birth status, as calculated using gestational age from last menstrual period and estimated due date, and classified those with measured birth weight greater than or equal to the 95th percentile for weight at 36 weeks gestational age as term. We did not report ACS use by gestational age, acknowledging the uncertain quality of the assessment.

The ACS rates are reported for all <5th percentile babies and by subgroups according to the location at delivery: hospitals, clinics (health centers), and the community (home). The hospitals were further subdivided into those having cesarean section and neonatal care capabilities (bag and mask and oxygen or mechanical ventilation). For this subgroup analysis, we limited the dataset to births that either occurred at home or at a facility that was regularly utilized by the women living in the MNH clusters. For each health facility, we determined whether or not each of the following services had been provided to at least two people during the course of the trial: cesarean section, bag and mask resuscitation, and oxygen or mechanical ventilation. Deliveries were then divided into those occurring at a facility with all of these care capabilities versus at home or a facility with none or some of the capabilities. We also describe the antepartum complications generally associated with risk of preterm delivery and indicators of use of ACS reported for the mothers of <5th percentile babies. Additionally, we report the characteristics of all women and babies delivered at the control clusters by site. All analyses were done with SAS v 9.3 (SAS Institute, Cary, NC).

## Results

From our survey, all sites except one reported that guidelines for ACS at the hospital level of care were routinely available. Additionally, several sites (Guatemala, Belgaum, India, and Pakistan) had guidelines for prescribing ACS at the primary health centers and only Pakistan had guidelines to administer ACS at the community level. The recommended gestational age (GA) for ACS use varied, but generally included administration of ACS for women with signs of preterm birth at 28 to 36 weeks. All sites reported that only doctors or medical officers could prescribe ACS at hospitals or health centers with the exception of Zambia and Pakistan, which allowed ACS prescription by nurses. All sites reported that prescribed ACS could be administered by doctors or nurses.

During the trial period, 51,523 women delivered at the control clusters across all sites, with the number of deliveries ranging from 2,292 in Argentina to 16,630 in Belgaum, India (Table 1). In Argentina, 99 % of births occurred in hospitals, compared with 75 % at the Indian sites and 14 % in Kenya. Among all births, rates of <5th percentile neonates for the site including both stillbirths and live births ranged from 3.5 % in Kenya to 10.7 % in Pakistan. The estimated rates of preterm birth ranged from 7.8 % in Guatemala to 14.8 % in Pakistan. Low birth weight (LBW, <2500 g) rates ranged from 3.5 % in Kenya to 20.3 % in Pakistan.

**Table 1:** Characteristics of the ACT control clusters by site during the ACT trial period

	Latin America		South Asia			sub-Saharan Africa	
	Argentina	Chimaltenango, Guatemala	India, Belgaum	India, Nagpur	Thatta, Pakistan	Kafue, Zambia	W. Provence, Kenya
Clusters, N	6	9	20	20	20	10	16
Total deliveries, N	2,292	3,960	16,630	7,597	8,202	5,739	7,103
Birth location							
Hospital	2,275 (99.3)	1,753 (44.3)	12,374 (74.4)	5,828 (76.7)	3,261 (39.8)	864 (15.1)	990 (13.9)
Clinic	3 (0.1)	45 (1.1)	3,374 (20.3)	1,648 (21.7)	1,758 (21.4)	2,833 (49.4)	2,014 (28.4)
Home	13 (0.6)	2,162 (54.6)	882 (5.3)	119 (1.6)	3,182 (38.8)	2,042 (35.6)	4,099 (57.7)
Birth attendant							
Physician	1,847 (80.7)	1,784 (45.1)	11,123 (66.9)	5,956 (78.4)	2,226 (27.1)	146 (2.5)	151 (2.1)
Nurse/Midwife	430 (18.8)	19 (0.5)	4,733 (28.5)	1,537 (20.2)	2,269 (27.7)	3,462 (60.3)	2,916 (41.1)
TBA	0 (0.0)	2,133 (53.9)	175 (1.1)	48 (0.6)	3,565 (43.5)	1,322 (23.0)	3,191 (44.9)
Family/Self Delivery	12 (0.5)	24 (0.6)	599 (3.6)	56 (0.7)	141 (1.7)	809 (14.1)	845 (11.9)
Antenatal care (≥1)	2,162 (94.6)	3,924 (99.2)	16,459 (99.9)	7,585 (99.9)	7,231 (89.7)	5,711 (99.6)	6,999 (98.7)
Methods to estimate delivery date							
LMP only or LMP with clinical exam	518 (22.8)	2,994 (75.6)	14,444 (86.9)	7,306 (96.2)	5,504 (67.1)	5,619 (98.0)	6,333 (89.4)
Clinical exam only	28 (1.2)	87 (2.2)	203 (1.2)	28 (0.4)	133 (1.6)	0 (0.0)	22 (0.3)
USG only or USG with other	789 (34.7)	636 (16.1)	364 (2.2)	16 (0.2)	616 (7.5)	0 (0.0)	0 (0.0)
Not applicable (after delivery)	854 (37.5)	165 (4.2)	1,610 (9.7)	235 (3.1)	316 (3.9)	71 (1.2)	366 (5.2)
Date unknown	87 (3.8)	77 (1.9)	9 (0.1)	7 (0.1)	1,629 (19.9)	45 (0.8)	365 (5.2)
Preterm births	184 (9.4)	308 (7.8)	1,743 (10.4)	714 (9.3)	1,098 (14.8)	667 (11.6)	615 (8.9)
<2500 g birth weight	163 (7.1)	514 (12.9)	3,216 (19.2)	1,143 (14.9)	1,684 (20.3)	301 (5.2)	252 (3.5)
<5th percentile for birth weight babies	149 (6.4)	192 (4.8)	874 (5.2)	377 (4.9)	890 (10.7)	263 (4.5)	252 (3.5)

Table includes pregnancies resulting in stillbirths and live births. *TBA* traditional birth attendant, *USG* ultrasonography

Among the risk factors reported for mothers who delivered <5th percentile babies, the two most common were threatened preterm labor and preterm premature rupture of membranes (PPROM) (Table 2). Hypertensive disorders were reported to be much higher in Pakistan than the other sites, as were reported cases of chorioamnionitis or fever (37.6 %). The most common type of ACS used across all sites was dexamethasone. A full course of ACS (considered to be four doses for dexamethasone or two doses for betamethasone) was only given to 18 % of women with <5th percentile babies in Argentina and was less than 3 % in all other sites.

**Table 2:** Signs of risk of preterm birth and ACS use among mothers of small babies (stillbirths and livebirths) by site during the trial period

	Latin America		South Asia			sub-Saharan Africa	
	Argentina	Chimaltenango, Guatemala	India, Belgaum	India, Nagpur	Thatta, Pakistan	Kafue, Zambia	W. Provence, Kenya
Women with small (<5th %ile) babies and non-missing, N	142	181	808	336	816	243	199
Antepartum conditions reported^a^							
Threatened preterm labor	21 (14.8)	76 (42.0)	507 (62.7)	115 (34.2)	231 (28.3)	83 (34.2)	38 (19.1)
Preterm PROM	17 (12.0)	33 (18.2)	127 (15.7)	31 (9.2)	299 (36.6)	42 (17.3)	22 (11.1)
Hypertensive disorders	20 (14.1)	24 (13.3)	96 (11.9)	10 (3.0)	267 (32.7)	4 (1.6)	13 (6.5)
Hemorrhage	8 (5.6)	5 (2.8)	54 (6.7)	6 (1.8)	155 (19.0)	31 (12.8)	34 (17.1)
Chorioamnionitis or fever	4 (2.8)	15 (8.3)	32 (4.0)	1 (0.3)	307 (37.6)	1 (0.4)	13 (6.5)
Other antepartum condition	14 (9.9)	2 (1.1)	46 (5.7)	11 (3.3)	79 (9.7)	7 (2.9)	3 (1.5)
No condition reported	73 (51.4)	85 (47.0)	208 (25.7)	196 (58.3)	157 (19.2)	122 (50.2)	113 (56.8)
ACS administered prior to delivery^a^	61 (44.2)	21 (12.0)	89 (11.0)	15 (4.7)	29 (3.6)	8 (3.4)	4 (2.0)
Dexamethasone	41 (29.7)	19 (10.9)	48 (5.9)	6 (1.9)	18 (2.2)	7 (3.0)	0 (0.0)
Betamethasone	17 (12.3)	0 (0.0)	28 (3.5)	4 (1.3)	0 (0.0)	0 (0.0)	0 (0.0)
Other steroids	0 (0.0)	0 (0.0)	3 (0.4)	0 (0.0)	1 (0.1)	0 (0.0)	0 (0.0)
Full course	25 (18.1)	3 (1.7))	19 (2.4)	0 (0.0)	3 (0.4)	6 (2.5)	0 (0.0)

Table includes pregnancies resulting in stillbirths and live births. Excludes 66 mothers of small babies who are missing data (2 from Argentina, 17 from Nagpur, 18 from Pakistan and 29 from Kenya)

^a^Denominator includes responses of ‘Yes’, ‘No’, ‘Don’t know’ and ‘Missing’

Use of ACS among mothers of <5th percentile babies was 44 % in Argentina, compared with 5–11 % in the Indian sites, 2–3 % in African sites and 12 % in Guatemala (Table 3). The same pattern was found when we used different categories such as preterm birth and LBW (<2500 g) to identify mothers that were eligible to receive ACS (data not shown).

**Table 3:** Antenatal corticosteroid use by level of care

	Latin America	South Asia	sub-Saharan Africa				
	Argentina	Chimaltenango, Guatemala	India, Belgaum	India, Nagpur	Thatta, Pakistan	Kafue, Zambia	W. Provence, Kenya
Women with small (<5th %ile) babies and ACS use reported, N	142	181	808	336	816	243	199
Received any antepartum steroids by location of delivery^1^							
Hospitals (overall), n (%)	61/137 (44.5)	21/94 (22.3)	80/645 (12.4)	14/252 (5.6)	21/342 (6.1)	7/63 (11.1)	2/53 (3.8)
Facilities with maternal and neonatal care capabilities^2^	57/131 (43.5)	16/62 (25.8)	40/198 (20.2)	12/163 (7.4)	12/214 (5.6)	0/0 (0.0)	1/25 (4.0)
Clinic, n (%)	0/0 (0.0)	0/3 (0.0)	8/95 (8.4)	1/43 (2.3)	7/162 (4.3)	1/101 (1.0)	1/62 (1.6)
Home, n (%)	0/1 (0.0)	0/78 (0.0)	1/67 (1.5)	0/23 (0.0)	1/312 (0.3)	0/72 (0.0)	1/84 (1.2)

^1^Denominator includes all births at facilities typically utilized by MNH participants and home births. ^2^Care capabilities include cesarean section, neonatal bag and mask, and neonatal oxygen or mechanical ventilation. It is further limited to those with data available on steroid administration

When we restricted the analysis to hospital births, the use of ACS in mothers of <5th percentile babies ranged from 4 % in Kenya to 45 % in Argentina. Sites in sub-Saharan African and south Asian countries showed ACS rates not higher than 13 %. Considering the use in hospitals with all maternal and neonatal care capabilities, the rates were slightly higher in Guatemala (25.9 %) and Belgaum, India (20.2 %). Zambia did not have any facilities with all care capabilities available; however, in all hospitals, 11 % of mothers with <5th percentile babies received ACS. In health centers, ACS were only used in South Asia, with the highest rate of 8.4 % found in Belgaum, India.

## Discussion

Across seven sites in LMICs, we found an overall low utilization rate of ACS among mothers of <5th percentile babies, ranging from 44 % in Argentina to 2 % in Kenya. In hospitals in particular, the rates in the sub-Saharan African and south Asian sites did not exceed 13 or 20 % when only hospitals with maternal and neonatal care capabilities were considered. With the exception of Argentina, these rates are lower than previous studies including only large hospitals with cesarean section capabilities [21].

One of the study limitations was that the survey was administered only at Global Network study sites and was not designed to be representative of the whole country. For the study data, because gestational age dating was unreliable, we used birth weight <5th percentile as a proxy measure. This decision may have misclassified some preterm births as term births. We also acknowledge that the subgroup of <5th percentile babies included around 40 % of small term babies. This misclassification could have resulted in an underestimation of the real use of ACS in preterm babies. Conversely, because the ACS utilization rates were measured in the context of a cluster randomized trial intending to increase its use, it is possible that the ACS rates observed in the control group in this study could have been actually higher than those seen in routine practice. In that sense, the ACS use rates in routine care may be even lower.

Our data showed that across all sites, between 1 and 9 % of <5th percentile babies born in clinics received ACS. In light of the current WHO preterm guidelines, and until evidence of benefit in those sites is available, this use should be discouraged. Considering these results, the recommendations in India, Pakistan and Guatemala to use ACS in women at high risk of preterm birth attending primary health centers should probably be revised.

The observed use of ACS among pregnancies likely to be preterm and occurring in hospitals is lower than 25 % in all sites except Argentina where ACS use is still lower than 50 %. These results may be interpreted in different ways. In hospitals that meet the conditions for ACS use based on the new WHO preterm guidelines, low rates of ACS use can be interpreted as substandard care. We have shown that the ACS rates in hospitals having cesarean section capacity and neonatal care including resuscitation and oxygen therapy are not substantially different than those reported for all hospitals. This low use would imply the need for improvement in use in such hospitals. However, this interpretation should be taken cautiously since our categorization of hospitals according to maternal and neonatal capabilities may not include the care actually available 24 h a day, 7 days a week. Moreover, this classification does not consider the capacity of the hospital to assess gestational age accurately.

## Conclusion

In hospitals with maternal and neonatal care capabilities meeting the WHO preterm guidelines criteria, ACS use is likely to be lower than expected. However, it is also likely that the majority of the health facilities included in this report did not meet these WHO criteria. As such, it is unknown if its use in these low resource hospitals results in more benefit than harm.
